# Actinic Cheilitis — From Risk Factors to Therapy

**DOI:** 10.3389/fmed.2022.805425

**Published:** 2022-02-15

**Authors:** Alina Vasilovici, Loredana Ungureanu, Lavinia Grigore, Elena Cojocaru, Simona Şenilă

**Affiliations:** ^1^Department of Dermatology, Iuliu Haţieganu University of Medicine and Pharmacy, Cluj-Napoca, Romania; ^2^Department of Dermatology, Cluj-Napoca Emergency County Hospital, Cluj-Napoca, Romania; ^3^Iuliu Haţieganu University of Medicine and Pharmacy, Cluj-Napoca, Romania; ^4^Carola Davila University of Medicine and Pharmacy, Bucharest, Romania; ^5^Department of Dermatology, Municipal Clinical Hospital, Cluj-Napoca, Romania; ^6^Department of Dermatology, Oradea Emergency County Hospital, Oradea, Romania

**Keywords:** actinic cheilitis, treatment, confocal microscopy, dermoscopy, risk factors, diagnosis

## Abstract

Actinic cheilitis or solar cheilosis is considered a precursor of malignancy or even an *in situ* squamous cell carcinoma (SCC) of the lip, located most frequently on the lower lip. Actinic cheilitis (AC) has a higher likelihood of developing into invasive SCC of the lip, which is one of the deadliest non-melanoma skin cancers. Risk factors include chronic UV exposure, increasing age, male gender, fair phototypes, chronic scarring, immunosuppressive therapy, and tobacco use. From a clinical point of view, AC is characterized by dryness, scaling, atrophy, indistinct borders, and erosions. Ulceration and the appearance of a nodule often suggest the progression to invasive SCC. Dermoscopic examination reveals white structureless areas, scales, erosions, and white halos of the vermilion. Reflectance confocal microscopy shows disruption of the stratum corneum, parakeratosis, an atypical honeycomb pattern, solar elastosis, and dilated and tortuous blood vessels with increased blood flow. The rate of malignant transformation ranges from 10 to 30% and early diagnoses and treatment are essential in preventing the development of invasive SCC. Optimal treatment has not been established yet, but invasive and topical treatments can be tried. The present paper aims to review the existing data regarding epidemiology, risk factors, clinical picture, non-invasive imaging, diagnosis, and therapy in AC.

## Introduction

Squamous cell carcinoma of the lower lip is a highly aggressive non-melanoma skin cancer, which composes almost 1/3 of all oral cancers. Most frequently, squamous cell carcinoma (SCC) of the lower lip begins as a precursor, actinic cheilitis (AC), or solar cheilosis (SC) (1). The rate of malignant transformation of AC into invasive SCC is higher than the rate for actinic keratosis; varies between 10 and 30% (2). However, up to 95% of SCCs on the lip occur on preexisting ACs (2). The metastasis rate is, moreover, four times higher in SCC of the lower lip compared with cutaneous SCC (3). As a consequence, early detection and early and correct treatment are essential for prognosis. Fortunately, the incidence of SCC is lower on the lip than on the cutaneous surface (1).

## Risk Factors

The most important risk factor for AC is chronic sun exposure (1). Fair skin, increasing age, occupation, and leisure activities involving intense sun exposure, the geographic latitude of residence, male gender, genetic predisposition, and immunosuppression are additional risk factors (1). A causative relationship between AC or SCC of the lip and tobacco smoking has not been established. However, tobacco seems to play a role in the progression of AC to SCC although it does not induce AC on its own (1). Alcoholism, chronic scarring, poor oral hygiene, and organ transplantation are other risk factors that may increase the severity of AC, favoring the development of invasive carcinoma (1).

In a cross-sectional, multicenter study from northwest Spain, Rodriguez-Blanco et al. showed that age over 60 years, skin phototype II, working outdoor for more than 25 years, and personal history of non-melanoma skin cancers were independent risk factors for AC (1). Spending more than 4 h/day in the sunlight was another risk factor (4). Another study found that patients with a lower level of education and those who used pesticides were more prone to develop AC (5).

The study of Rodriguez-Blanco et al. showed an association between different risk factors and clinical manifestations (6). Thus, desquamation and erythema were associated with working outdoors for more than 25 years and high alcohol intake. Smoking was associated with less erythema, while a mottled appearance was associated with a history of non-melanoma skin cancer.

The study conducted by Santos et al. conducted a study in a group of extractive mining workers from Brazil and found a correlation between clinical presentation and time spent outdoors. Those who spent more than 15 years in the sun had a more aggressive appearance (characterized by atrophy, vague delimitation between the vermilion and the skin, fissures, mottled appearance, indurated lip, and ulceration), and those with less than 15 years of sun exposure presented edema, erythema, xerosis and scaling, brown spots, soft lip, and involvement of less than ½ of the lip (7).

## Clinical Picture

**Figure 1:**
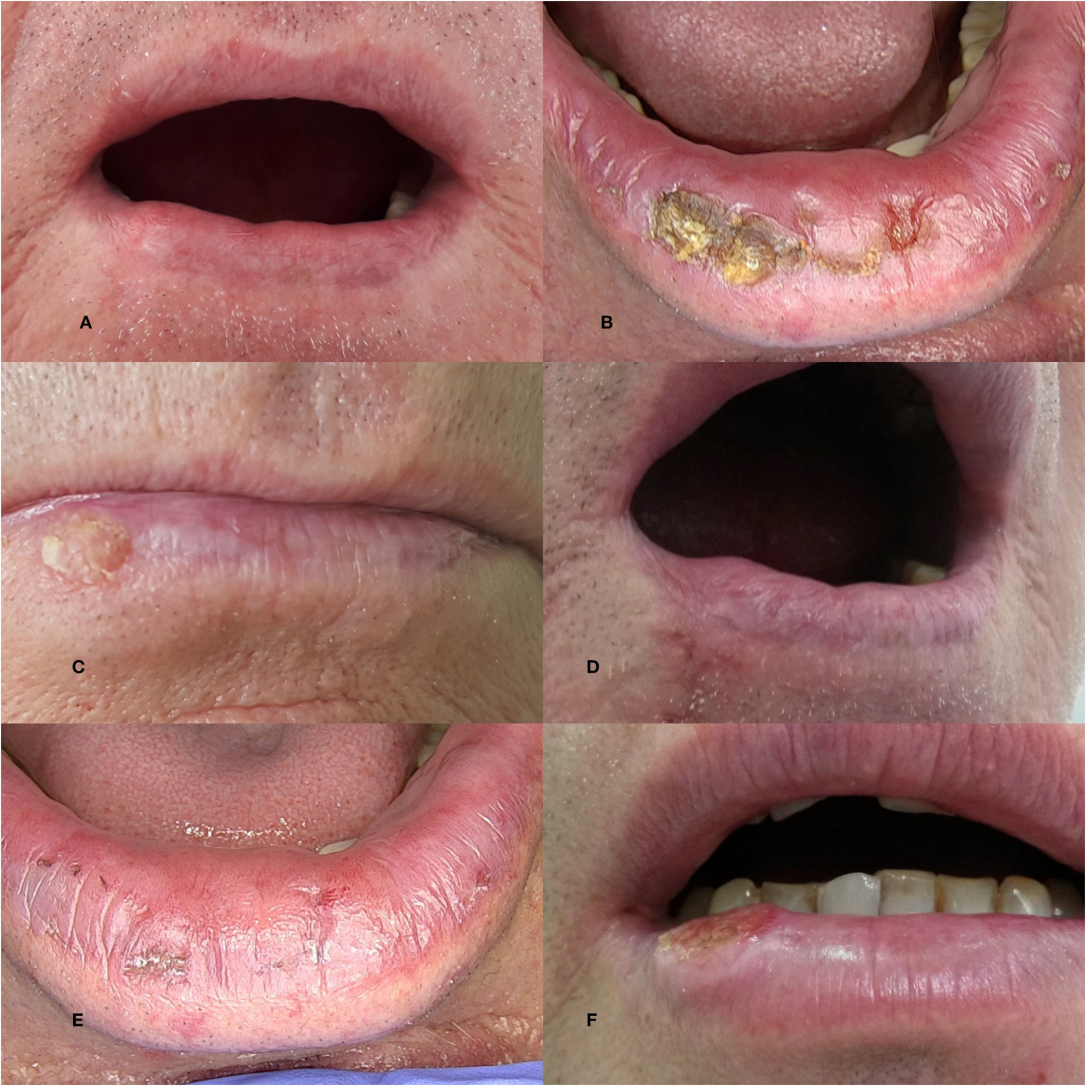
The clinical picture of actinic cheilitis (AC) C: **(A)** Xerosis; **(B)** Hyperkeratosis; **(C)** Mottled appearance; **(D)** Blurred demarcation of the vermilion border; **(E)** Fissures; **(F)** Plaque.

Actinic cheilitis (AC) is considered a premalignant or a SCC *in situ* located on the vermillion or semimucosa of the lips, most often on the lower lip. It is a chronic alteration of the lips and the clinical picture can vary widely and there is no generally accepted classification of the disease. The AC may be similar to the classic form of actinic keratosis with well-demarcated, erythematous papules, or plaques with scale; but often there is a diffuse, multifocal, and heterogeneous lesion characterized by xerosis (Figure 1A), scales, hyperkeratotic areas (Figure 1B), and even atrophy. The atrophy was defined as the depression of the lip that results from the thin epidermis/dermis (8). At palpation, it feels like sandpaper. The color of the lip can be changed: erythema (explained by vasodilatation), spotting (change of color of the normal mucosa without elevation or depression), a mottled appearance (erythema and white patches) (Figure 1C), or brown spots (7, 8). The demarcation between the lip and the surrounding skin can be blurred (Figure 1D). There can be loss of tissue presenting as vertical fissures (linear clefts extending into the dermis) (Figure 1E) or ulceration (full thickness loss of the epidermis) (8). A plaque (Figure 1F) can be found (described as a raised, flat lesion, > 1 cm diameter) or the entire lip can be indurated (7, 8). Areas of leukoplakia can be seen.

Usually, AC is asymptomatic, but some patients accuse lip dryness, cracking of the lips, a burning or stinging sensation, pain, or even abnormal lip mobility (2). The skin phototype of the majority of patients is Fitzpatrick I-II (1).

The study of Poitevin et al. proposed a clinical score for AC, comprising 4 grades, in which the first one represents the onset of the disease, and the last one is the progression of the disease to an invasive SCC. Grade I was defined by xerosis and desquamation of the vermilion; grade II included atrophy (palid areas and eruptions and soft superficies) and a blurred demarcation between lip and skin or a melanotic line as the vermilion limit. Grade III was characterized by squamous and hyperkeratotic areas extending into the wet mucosa. Grade IV involved ulceration (single or multiple) or leukoplakia, together with indurated areas, suggesting a malignant transformation (9).

In a study conducted by Rodriguez-Blanco et al. AC was defined by these characteristics: long-term desquamation, unremitting erythema, a mottled image (red and white patches), a plaque, and an erosion/ulceration. Almost half of the patients (47.3%) had just one clinical characteristic, 40.2% - 2, 12.3% - 3, and 0.2% presented 4 modifications. A mottled appearance was encountered most often (73.8%), accompanied by desquamation (53.7%) and erythema (30.1%). A plaque and an ulceration were found in less than 5% of patients. Other signs of actinic damage (actinic keratosis/lentigines) were found in 73.5% of patients (6).

The main differential diagnosis for AC is lichen planus with lesions on the lip. Czerninski et al. compared the clinical features of these diseases and found that lip lesions of lichen planus were white, more limited, and symptomatic, while the AC was more extensive, diffuse, and was composed of a mixture of red and white areas, the patients being disturbed by the aspect of their lower lip (10). Other differential diagnoses are inflammatory disorders (eczema, leukoplakia, and cheilitis granulomatosa), or just lip xerosis with chronic irritation (2). The confusion with “dry lips” is the reason for which, generally, the patient postpones the medical consultation.

### Dermoscopy

The diagnosis of AC is often made clinically, and dermoscopic examination is not performed regularly before treatment. Therefore, there is scarce information in the literature about the dermoscopic features of the disease.

**Figure 2:**
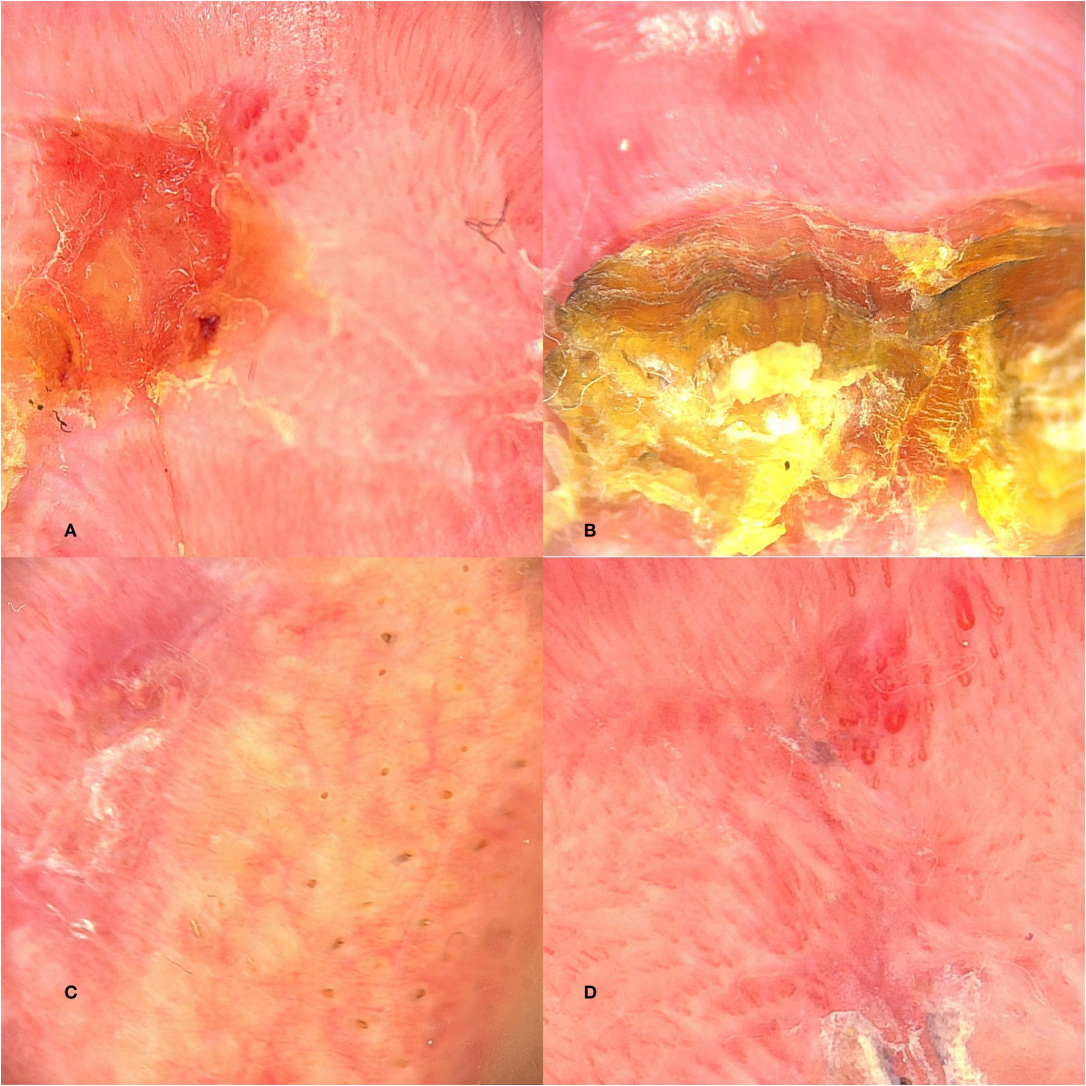
Dermoscopic features of AC: **(A)** Structureless areas; **(B)** Scales; **(C)** White halo of the vermilion border; **(D)** Telangiectatic and torturous blood vessels.

White structureless areas (Figure 2A), scales (Figure 2B), and white halo of the vermilion border (Figure 2C) are described frequently (11, 12). The blood vessels are telangiectatic and tortuous (Figure 2D), but sometimes polymorphous vessels can be seen (2). Island-like structures with white projections and vascular telangiectasia disposed radially to an ulcerated area were described by Ito et al. (11). The ulcerated lesion is covered by a crust described by dermatoscopy as a brown structureless area.

In one study, dermoscopy was used to evaluate the response of a topical treatment for AC. At 7 days, post-treatment, the dermoscopic examination revealed erythema, edema, scales, erosions, and crusts, and complete recovery of the lip after 30 days (12).

### Reflectance Confocal Microscopy

Reflectance confocal microscopy (RCM) is a non-invasive imaging technique that allows examination of the epidermis and superficial dermis at resolutions almost similar to those used in optical light microscopy (2). The epidermis of the lips is thinner than in other areas of the body, making the lips an optimal site for RCM examination and, thus, contributing to the early detection of malignant transformation for AC.

Reflectance confocal microscopy (RCM) features for AC is described in the epidermis and superficial dermis. The stratum corneum is disrupted with single detached cells; hyperkeratosis and parakeratosis can be seen. At stratum granulosum and spinosum, an atypical honeycomb pattern is described with cells and nuclei of various sizes and shapes. In the superficial dermis, there is solar elastosis defined as bright dense bundles that resemble a lace; the blood vessels are dilated and tortuous with the increased flow; inflammatory cells can be observed. The most important RCM characteristics for the diagnosis of AC are atypia of the keratinocytes and the atypical honeycomb pattern (2, 13).

In a study about RCM diagnostic criteria for AC and SCC, Lupu et al. showed that the atypical honeycomb pattern and the presence of the targetoid cells in the epidermis were strongly associated with AC. Hyperkeratosis, ulceration, and dermal solar elastosis were features found both in AC and SCC. The blood vessels diameter was larger in SCC than in AC (14).

Reflectance confocal microscopy (RCM) can also be used to follow the outcome after local treatment. Benati et al. showed a regular honeycomb pattern with uniform cells at 30 days after topical therapy with ingenol mebutate gel (12).

### Malignant Transformation of AC

Actinic cheilitis (AC) is defined as a precursor condition that may evolve to an SCC. The malignant transformation varies from 10 to 30% (15), but it is reported that 95% of SCC of the lower lip develop on a preexisting AC (16). The SCC of the lower lip is more aggressive than the SCC of the skin, with a four-time higher metastasis rate (16).

Without treatment, the AC progresses slowly, while patients neglect the lesion and misinterpret it as a sign of aging. The progression was described to happen from 1 to 30 years (17). Clinically, when there are erosions or ulcerations, a nodule, or bleeding - the progression to SCC should be suspected (15) and a biopsy or excision performed.

The study of Markopoulos et al. followed up with the patients with AC for 0.3–10 years and reported two cases of progression to SCC after 2.4 and 2.8 years, leading to an overall malignant transformation rate of 3.07% (18).

The malignant transformation of AC after different treatments was studied. Low rates (2.33 and 7.14%) of malignant transformation were observed after CO2 laser vermilionectomy. In studies where patients were treated with non-invasive methods, there was no disease progression during the follow-up period (1.5–4 years) (16).

Thus, the patients with AC should be followed up periodically and incisional biopsies should be performed from clinically suspicious lesions to hinder their malignant transformation, but there is no general consensus regarding the period of time needed for follow-up.

### Therapy

Due to the high rate of transformation into invasive SCC, early diagnosis and treatment are essential for prognosis in AC. However, due to the particular anatomical and histological characteristics (proximity of the mucosa and thin skin), as well as the importance of the cosmetic outcome, treatment is difficult, and there is no general consensus regarding the best therapeutic approach (3). Moreover, an accepted clinical tool for the measurement of the severity and therapeutic response in AC is lacking. Clearance can be assessed based on clinical, dermoscopic, or histologic criteria, recurrence, or progression rate. Side effects, patient satisfaction, or cosmetic outcome are other important criteria that must be considered (16).

Treatment options include surgical procedures (vermilionectomy, cryotherapy, laser ablation, and Mohs surgery), conservative modalities (topical treatment using imiquimod, 5-fluorouracil, diclofenac, ingenol mebutate), and photodynamic therapy (3, 16, 19–21).

## Surgical Treatments

The most frequently used surgical treatment is vermilionectomy, a radical procedure that consists of the complete removal of the lip mucosa. This procedure is associated with the highest rate of complete response, the recurrence rate after vermilionectomy being very low (3, 20). More than 80% of patients with histopathological assessment achieved complete response (16). Side effects include swelling, bruising, paresthesia, infection, necrosis, hematomas, and difficulty eating immediately after the treatment (3, 16, 20, 21). Cosmetic outcome was described as excellent by most of the patients, and one study comparing W-plasty to classic vermilionectomy has found less scar retraction in patients treated with W-plasty (3, 22).

Laser ablation, using carbon dioxide laser or erbium-doped yttrium aluminum garnet (Er-YAG) laser, showed 93.8% complete clinical response, and 96.1% complete histological response (3, 21). A clinical recurrence rate of approximately 6% was reported (16, 21). Difficulty eating, bleeding, and edema were the most frequently described adverse events, but the number of reported adverse events per patient is very low (0,42/case). The cosmetic outcome was excellent, with no scarring in all cases (3, 16, 21).

Electrodesiccation leads to clinical improvement in most of the patients, but the histological response is relatively low. Pain and burning sensation are the most frequently reported side effects (23).

### Conservative Modalities

Imiquimod 5% leads to a clinical improvement in 80–100% of the cases, complete histological regression in 73% of the cases, and studies that assessed the degree of histological dysplasia showed decreased in dysplasia in all cases (20). The number of adverse events per case was 3.1 and was represented by edema, pain, and ulceration during treatment (3, 16).

The 5-fluorouracil (FU) was conducted to complete the clinical response in 75% of patients, interestingly 1% FU demonstrating a better performance compared to 5% FU. However, the recurrence rate was significant (31.8%) (3). Moreover, interruptions due to adverse events were reported in 10% of the cases, the most important ones being pain, irritation, and difficulty to eat and speak (20).

When diclofenac gel was used, 45.16% of patients experienced a complete clinical response, while in studies that assessed the histopathological response, complete remission was observed in 66.67% of patients. The reported recurrence rate was 6.52%, and the aesthetic result was rated as excellent by all patients (16). The reported side effects were erythema, edema, and burning sensation, and the discontinuation due to adverse events was up to 15.22% (16, 24).

Ingenol mebutate was assessed in 2 studies and its application led to a complete remission in 41.18% (12, 16, 25). No recurrence was described during follow-up. All patients presented side effects: erythema, erosions, vesicles, scales, and crusts; but none of them discontinued the treatment (16).

### Photodynamic Therapy (PDT)

The improvement rate ranged between 47 and 100% when photodynamic therapy (PDT) was used, in which, the complete clinical response was reported in 68.9% of cases (20, 21). The variant of treatment using daylight showed better results, and ALA-PDT performed better than MAL-PDT. The histopathological response was complete in almost half of the cases (49.48%), with higher response rates for ALA-PDT (16). The most common side effects reported were: erythema, edema, pain, and crusts, with an estimated number of side effects per patient of 2.4. A percentage of 5.86% of patients discontinued the therapy due to adverse events. The recurrence rate was 14.07% (16). The cosmetic result was considered excellent in 67.65% of patients (16).

## Conclusions

Actinic cheilitis is considered a premalignant lesion, or an *in situ* SCC of the lip, with a high likelihood of developing into invasive SCC. Risk factors include chronic UV exposure, increasing age, male gender, fair phototypes, chronic scarring, immunosuppressive therapy, and tobacco use. Clinically, AC is frequently described as a white and scaly plaque with indistinct borders and a sandpapery feel on palpation. Dermoscopic examination and reflectance confocal microscopy both help in the diagnosis and follow-up. The rate of malignant transformation ranges from 10 to 30% and early diagnoses and treatment are important to prevent the development of invasive SCC. Even though there are many treatment modalities available for AC, no general consensus regarding the proper management of AC exists and randomized clinical trials are needed to add more powerful evidence. However, invasive treatments seem to be the most effective in achieving a complete response, with a good safety profile.

## Author Contributions

LG and EC collected the literature. LU, AV, and SŞ conducted the literature review, manuscript drafting, and critical revision of the manuscript for important intellectual content. All authors contributed to the article and approved the submitted version.

## Conflict of Interest

The authors declare that the research was conducted in the absence of any commercial or financial relationships that could be construed as a potential conflict of interest.

## Publisher's Note

All claims expressed in this article are solely those of the authors and do not necessarily represent those of their affiliated organizations, or those of the publisher, the editors and the reviewers. Any product that may be evaluated in this article, or claim that may be made by its manufacturer, is not guaranteed or endorsed by the publisher.
